# Telmisartan in the diabetic murine model of acute myocardial infarction: dual contrast manganese-enhanced and delayed enhancement MRI evaluation of the peri-infarct region

**DOI:** 10.1186/s12933-016-0348-y

**Published:** 2016-02-05

**Authors:** Ildiko Toma, Paul J. Kim, Rajesh Dash, Michael V. McConnell, Dwight Nishimura, Phillip Harnish, Phillip C. Yang

**Affiliations:** Department of Medicine, Stanford University, Stanford, CA USA; Department of Electrical Engineering, Stanford University, Stanford, CA USA; Eagle Vision Pharmaceutical Corporation, Downingtown, PA USA; Cardiovascular Medicine, 300 Pasteur Drive, H2157, Stanford, CA 94305-5233 USA

**Keywords:** Manganese-enhanced MRI, Delayed-enhanced MRI, Peri-infarct injury, Diabetes mellitus, Telmisartan

## Abstract

**Background:**

A novel MRI technique, employing dual contrast manganese-enhanced MRI (MEMRI) and delayed enhancement MRI (DEMRI), can evaluate the physiologically unstable peri-infarct region. Dual contrast MEMRI–DEMRI enables comprehensive evaluation of telmisartan to salvage the peri-infarct injury to elucidate the underlying mechanism of restoring the ischemic cardiomyopathy in the diabetic mouse model.

**Methods and results:**

Dual contrast MEMRI–DEMRI was performed on weeks 1, 2, and 4 following initiation of telmisartan treatment in 24 left anterior descendent artery ligated diabetic mice. The MRI images were analyzed for core infarct, peri-infarct, left ventricular end-diastolic, end-systolic volumes, and the left ventricular ejection fraction (LVEF). Transmission electron microscopy (TEM) and real-time PCR were used for ex vivo analysis of the myocardium. Telmisartan vs. control groups demonstrated significantly improved LVEF at weeks 1, 2, and 4, respectively (33 ± 7 %*** vs. 19 ± 5 %, 29 ± 3 %*** vs. 22 ± 4 %, and 31 ± 2 %*** vs 18 ± 6 %, ***p < 0.001). The control group demonstrated significant differences in the scar volume measured by MEMRI and DEMRI, demonstrating peri-infarct injury. Telmisartan group significantly salvaged the peri-infarct injury. The myocardial effects were validated by TEM, which confirmed the presence of the injured but viable cardiomyocyte morphology in the peri-infarct region and by flow cytometry of venous blood, which demonstrated significantly increased circulating endothelial progenitor cells (EPCs).

**Conclusion:**

The improved cardiac function in ischemic cardiomyopathy of diabetic mice by telmisartan is attributed to the attenuation of the peri-infarct injury by the angiogenic effects of EPCs to salvage the injured cardiomyocytes. Dual-contrast MEMRI–DEMRI technique tracked the therapeutic effects of telmisartan on the injured myocardium longitudinally.

## Background

Diabetes mellitus currently afflicts 171 million people worldwide and its prevalence continues to rise [1, 2]. The adverse cardiovascular consequences of diabetes mellitus are recognized by the accelerated rate of atherosclerosis, predisposing patients to coronary artery disease, myocardial infarction, and death [3, 4].

Angiotensin-converting enzyme inhibitors (ACE) has been repeatedly shown to decrease cardiovascular mortality in the diabetic population [5]. Angiotensin-receptor blockers (ARB) were developed for patients intolerant of ACE inhibitors and have similarly shown to reduce major cardiovascular disease outcomes [6]. Telmisartan, in particular, has been shown to be equivalent to ACE inhibitors in providing cardiovascular protection in high-risk heart failure patients, particularly the diabetic population [7, 8]. It has been postulated that telmisartan may attenuate the myocardial injury in DM patients sustaining an MI. The anti-apoptotic, anti-fibrotic and angiogenic effects of telmisartan with the modulatory role on peroxisome proliferator-activated receptor gamma (PPARγ) may contribute to the microvasculature of the peri-infarct region to salvage the injured myocardium, reduce the remodeling of the myocardium and improve cardiac function [9].

The distinction between reversible and irreversible myocardial injury within the region at risk is imperative since the selection of an appropriate therapy can alter the overall mortality and morbidity significantly. However the distinction between the infarcted myocardium versus the injured but viable myocardium located in the peri-infarct region is often difficult to make [10]. The current gold standard is DEMRI, which employs a gadolinium (Gd^3+^)-based contrast agent using cardiac MRI. However, DEMRI does not provide direct cell viability information due to its non-specific distribution into the extracellular space and may overestimate the infarct region [11]. Thus dual-contrast MRI has been used to provide complementary information [12] and for the purpose of directly evaluating cell viability, DEMRI can be complemented by MEMRI [13, 14]. MEMRI employs Mn^2+^, an essential heavy metal ion that enters cells via voltage-gated calcium channels [15]. Only the viable cells are able to accumulate Mn^2+^ within the intracellular space, shortening the T1 relaxivity and increasing the MRI signal. MEMRI combines high spatial and temporal resolution of MRI with a viability-specific intracellular signal to identify viable cardiomyocytes [16, 17].

In this study, we performed a dual-contrast DEMRI–MEMRI to evaluate the peri-infarct region in a diabetic murine myocardial injury model. This complementary technique assessed the therapeutic effect of telmisartan on the peri-infarct region and correlated the reduced peri-infarct region with the resultant restoration of cardiac function [11].

## Methods

### Diabetic murine myocardial injury model

All animal studies were approved by the Stanford University Administrative Panel on Laboratory Animal Care. The C57BL/KLS-lepr^db^/lepr^db^ (*db/db,* Jackson Laboratories, Bar Harbor, ME, USA), a transgenic mouse strain with an autosomal recessive mutation in the leptin receptor, is a well-established animal model of type 2 diabetes mellitus [18]. Myocardial injury was induced in a total of 24 adult *db/db* mice. This provided a model of myocardial injury with an LVEF of 15–20 % in control groups as previously demonstrated [19]. Mice were anesthetized with inhalational isoflurane 1.25–2.0 % and subcutaneous buprenorphine 0.1 mg/kg. They were intubated to achieve positive pressure ventilation with oxygen/isoflurane mixture. Thoracotomy was performed and the left anterior descending coronary artery (LAD) was ligated until blanching of the distal left ventricle was seen. The chest was then closed in layers and the animal was placed in the small animal intensive care unit. The animals were randomly assigned to either the telmisartan-treated or control group. Telmisartan (10 mg/kg/day; Boehringer-Ingelheim Co., Ltd.) was administered per os in the drinking water 1 week after LAD ligation for 12 mice (telmisartan group) and tap water was available ad libitum for another 12 mice (control group). Telmisartan was dissolved in the drinking water and made fresh every 5 days. Five milliliters of telmisartan water was available per mouse daily. There were 2–3 mice from the same treatment group in each cage. During the treatment period, the telmisartan-treated animals were checked three times daily to make sure there was enough drinking water available for them. Telmisartan water was replenished each morning. When the telmisartan water ran out during the day, regular tap water was added to keep them hydrated. Imaging was performed an additional week after LAD ligation (i.e., 1 week *after* treatment). The corresponding weeks 1, 2 and 4 referred to in the manuscript refer to weeks after treatment. Due to severe myocardial injury in this already morbid mouse strain, we observed an increased mortality rate of approximately 75 % in both groups at the end of the study. There was no significant difference between the telmisartan and control groups in terms of mortality. At a minimum, the mice were monitored twice daily (in the early morning and late afternoon, including weekends and holidays) and any animals displaying clinically abnormal behavior were removed from the group and instead housed individually with ready access to food and water. Treatment for the telmisartan mice was continued throughout the study. Supportive care was provided in the form of water-soaked pellets placed on the cage floor, administration of subcutaneous fluids, and provision of a high calorie oral supplement (e.g., Diet Gel, Nutri-Gel, Nutrical). Regular mouse diet and dark/light cycle were provided to the animals. We did perform 3 additional MRI studies for week 2 for the control group to further increase the number and these numbers also contributed to pressure–volume analyses and in vitro tests at the conclusion of the study.

### In vivo MEMRI and DEMRI

Anesthesia was induced and maintained with 1.25–2.0 % isoflurane. ECG leads were inserted subcutaneously to assess the heart rate while the body temperature was maintained at 37 °C and the respiratory rate was monitored. Using a 3T GE Signa Excite scanner with a dedicated mouse surface receive-only coil (Rapid MR International, Germany), multiple cardiac functional parameters were obtained on weeks 1, 2, and 4 after initiation of telmisartan treatment. The following sequences were performed for MRI acquisitions: (1) MEMRI was performed using fast gradient echo inversion recovery (GRE-IR) sequence with FOV 4 cm, slice thickness 1 mm, matrix 256 × 256, TE 3.4 ms, FA 45°, 2R-R acquisition, TI 300-500 ms, and NEX2 with an intraperitoneal injection of 0.7 cc/kg of Eagle Vision Pharmaceutical manganese-based contrast solution (EVP1001-1, Eagle Vision Pharmaceutical Corp) prior to MEMRI acquisition; (2) DEMRI was performed 24 h later with an intraperitoneal injection of 0.2 mmol/kg gadopentetate dimeglumine (Magnevist, Berlex Laboratories) using a similar GRE-IR sequence but with a TI of 200-300 ms; and (3) cardiac MRI of LV volumes and function were performed on the day of MEMRI using fast spoiled gradient echo with FOV 5 cm, slice thickness 1 mm, matrix 256 × 256, TE 5 ms, and FA 30. Consecutive short axis slices were obtained for quantitative analysis for LV volumes and function and DEMRI and MEMRI volumes. For LV volumes and function assessment, 20 cardiac phases were obtained for each short axis slice and typically 5–7 short axis slices were obtained for each mouse. Injection of contrast was performed 30 and 10 min prior to image acquisition for MEMRI and DEMRI respectively. The optimal timing of contrast injection prior to image acquisition was previously determined by observation of peak signal to noise ratio for both MEMRI and DEMRI.

### MRI image analysis

For each short-axis slice, planimetry measurements of the LV myocardium were conducted off-line by manual tracing of the epicardial and endocardial borders at end-systole and end-diastole with OsiriX (Pixmeo Inc., Geneva, Switzerland). Manual tracings were not statistically different from semi-automatic full width half maximum tracings as demonstrated in a previous study [11]. The papillary muscles were considered part of the LV cavity. Left ventricular mass, LV end-diastolic volume (LVEDV), and LV end-systolic volume (LVESV) were measured to calculate the LVEF. For infarct analysis, the MEMRI defect area and the DEMRI enhanced area were designated as scar tissue. These areas were traced in short-axis slices, using cine images for comparison, and integrated to determine scar volumes by MEMRI and DEMRI in matched mice hearts (n = 24). The % MEMRI scar volume = (MEMRI defect volume/total LV volume) × 100 and % DEMRI scar volume = (DEMRI scar volume/total LV volume) × 100. The difference between MEMRI and DEMRI defect volumes defined the peri-infarct volume.

### Pressure–volume loop analysis

At the end of the study, ventricular performance was assessed by pressure–volume (PV) loop analysis with a 1.4F conductance catheter (Millar Instruments, Inc., Houston, TX) before the animals were sacrificed in 8 mice (telmisartan group, n = 4; control group, n = 4) [19]. The right carotid artery was cannulated with the Millar catheter that was advanced through the aortic valve into the left ventricle. The PV relations were measured at baseline and during inferior vena caval occlusion. When coupled with pressure, the generation of ventricular PV relationships allowed precise hemodynamic characterization of ventricular systolic and diastolic function and loading conditions. These data were analyzed with PVAN 3.4 Software and Chart/Scope software (ADInstruments, Inc., Colorado Springs, CO).

### Flow cytometry

Venous blood was obtained from the inferior vena cava at the end of the study for 8 mice (telmisartan group, n = 4; control group, n = 4). The whole blood was then centrifuged 5 min at 3000 rpm and the supernatant subsequently removed. The pellets were resuspended by equal volume of ACK lysis buffer (Lonza, Allendale, NJ) to remove red blood cells and centrifuged again. The pellets were washed and resuspended in 100 μL PBS with 5 % fetal bovine serum and stained with purified primary anti-human CD34 antibody (BD Pharmingen, San Diego, CA) and PE anti-mouse IgG secondary antibody. The cells were then evaluated by multi-color flow cytometry using FACScalibur (BD Biosciences, San Jose, CA) with subsequent analysis of data in FlowJo (Tree Star, Ashland, OR).

### Real-time PCR

Total mRNA was isolated from telmisartan-treated and control myocardial tissue at the end of the study (telmisartan group, n = 2; control group, n = 2). Total mRNA was then reverse-transcribed into cDNA. Real-time quantitative PCR was run on a 96 well real-time PCR thermocycler using Power SYBR Green master mix (AB, USA), according to the manufacturer’s recommendations. Primers for collagen I, collagen III, connective tissue growth factor (CTGF), TGFβ, fibronectin, and Akt were synthesized by Invitrogen (in the order of forward and reverse:

- collagen I: CCTGGAATGAAGGGACAC and GAGCTCCGTTTTCACCAG;
- collagen III: AAAAGGGTCCTCCCGGAGA and TTCCATCATTGCCTGGTC;
- CTGF: CAACCGCAAGATCGGAGT and GTCGGTAGGCAGCTAGGG;
- TGFβ: AAGCGCCCGGGTTGTGTTGG and CTGTACATTGACTTTAGG;
- fibronectin: TCTGTGCCACTTCCCCTT and ATACATGACCCCTTCATT; and
- Akt: TTGAGCGCACCTTCCATG, TTCATGGTCACACGGTGC). The GAPDH housekeeping gene (CCTCAAGGGCATCCTGGGCT, GCTGGTGGTCCAGGGGTCTT) was used as reference for the relative quantification of the genes of interest. Despite the small sample size, we previously were able to demonstrate statistical significance with similar numbers [19].

### Immunohistology and transmission electron microscopy

At the end of the study, explanted hearts were fixed with 4 % formalin and paraffin embedded and standard hematoxylin and eosin staining was performed on short axis sections.

For TEM, remote, peri-infarct and infarct regions were identified using Evan’s blue dye (EBD). EBD enters cardiac myocytes through injured sarcolemma and thus clearly identifies the infarct region. 90 μL of EBD was injected intraperitoneally, at a concentration of 10 mg per mL of PBS, 24 h prior to explantation. The following day, the myocardial tissue from a telmisartan-treated diabetic mouse 5 weeks post LAD ligation was obtained from the remote, peri-infarct and infarct regions and then cut into pieces (3–4 pieces per region) and fixed with 2 % glutaraldehyde and 4 % paraformaldehyde. The remote, peri-infarct and infarct zones were also co-localized with MEMRI and DEMRI images with the overlap of DEMRI and MEMRI enhancement identifying the peri-infarct region. Further fixation was performed with 1 % osmiumtetroxyde. After dehydration and embedding, sections were analyzed by a JEOL 1230 tissue electron microscope (JEOL Ltd., Tokyo, Japan) at 80 keV. Ultrastructural analysis of the cardiomyocytes was performed by blinded observers; assessing ‘healthy’ and ‘unhealthy’ features of cell integrity and sarcomeric organization [11]. For ‘healthy’ features, each cardiomyocyte was graded on whether it exhibited (Table: black): ‘5’—high abundance; ‘4’— moderate abundance; ‘3’—low abundance; ‘2’—rare; or ‘1’—complete absence of that feature. Conversely, for an ‘unhealthy’ TEM feature (Table: red): ‘5’ indicated that the nucleus displayed complete absence; ‘4’—rare; ‘3’—low abundance; ‘2’—moderate abundance; and ‘1’—high abundance of the ‘unhealthy’ feature. Individual scores from 10 to 15 myocytes per zone were averaged to generate an overall zone score for that feature from 1 to 5. The 17 scores were averaged to generate a composite score for each zone that reflected the overall structural integrity of the cells within each zone, with ‘5’ being the best and ‘1’ being the worst score. Agreement on TEM scoring between two independent observers was high (Kappa = 0.74). Composite scores were compared between remote zone, border zone, and core infarct zone TEM.

### Statistical analysis

Results are mean ± standard deviation. Comparison between groups was performed with the Wilcoxon rank-sum test. Significant differences (*p* < 0.05) among the TEM of the three regions (remote, intra-infarct and peri-infarct regions) were tested using one-way analysis of variance (ANOVA).

## Results

### Left ventricular systolic function, volume, and morphology

**Fig. 1:**
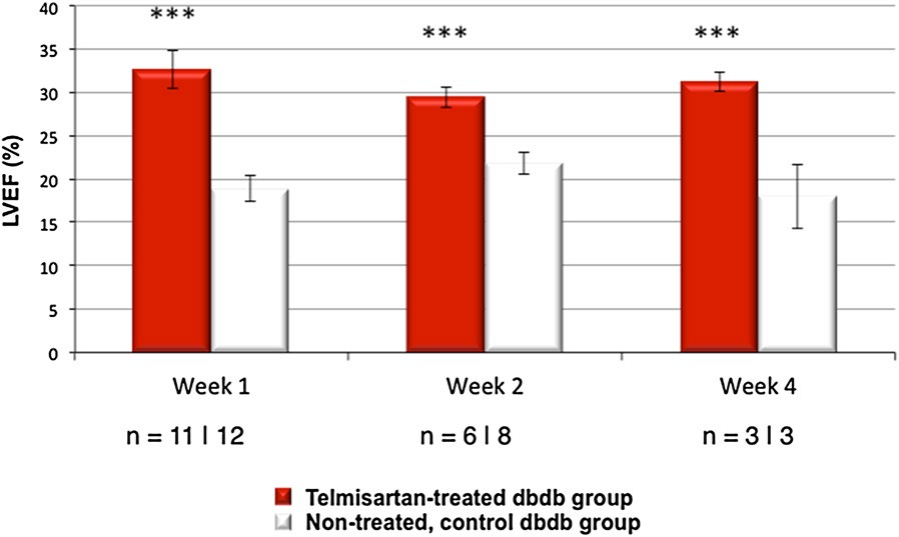
Functional measurement of telmisartan versus control groups. Significantly improved LVEF at weeks 1, 2, and 4 (***p < 0.001) in the telmisartan-treated (*red bar*) diabetic mice compared to control diabetic mice (*white bar*)

**Fig. 2:**
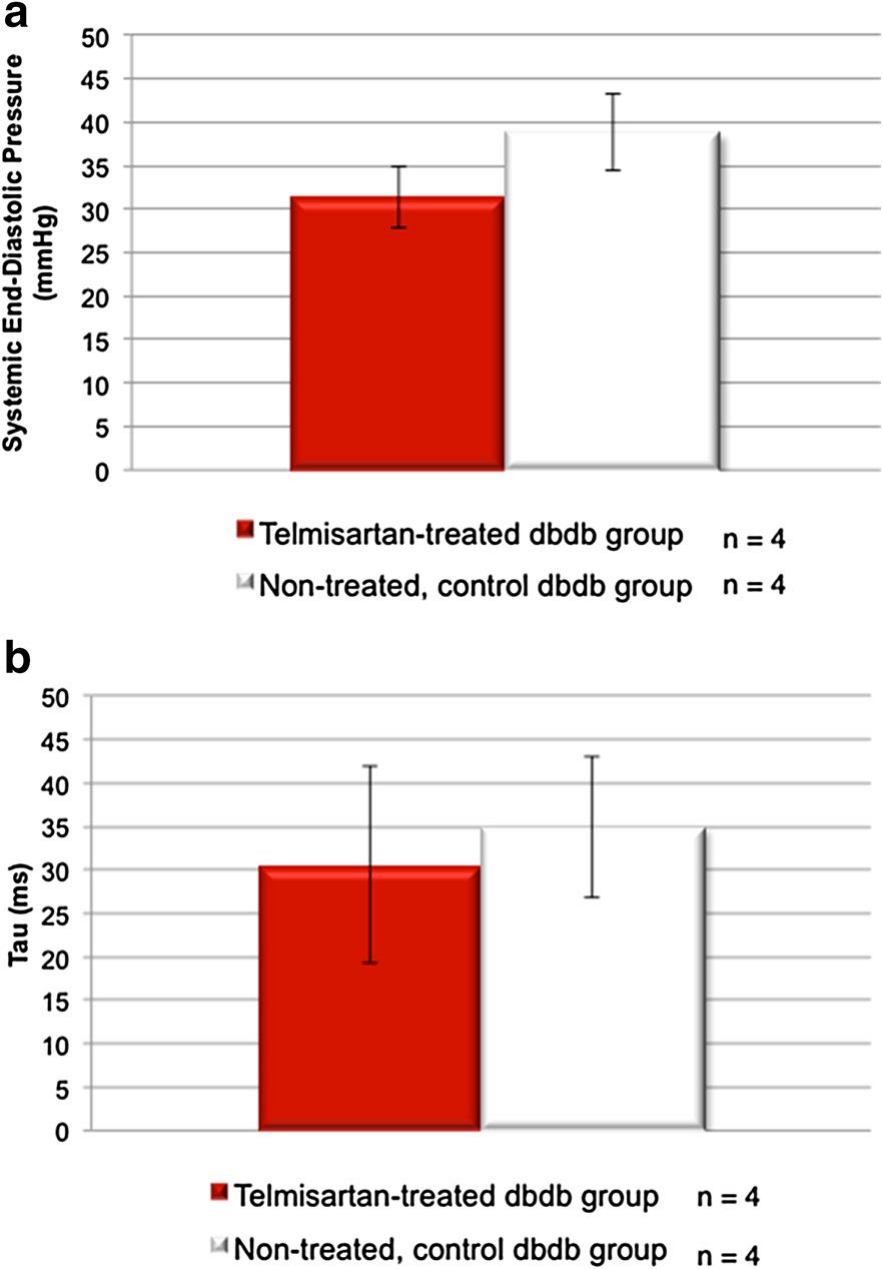
Invasive hemodynamic analysis. No significant difference is seen in the telmisartan-treated (*red bar*) diabetic mice compared to control diabetic mice (*white bar*) for both systemic end-diastolic pressure and the isovolumic relaxation constant, Tau, calculated by the Glantz method

At weeks 1, 2, and 4, LVEF was severely reduced (19 ± 5, 22 ± 4 and 18 ± 6 %, respectively) in the control group. However, the telmisartan group demonstrated significant and sustained increase in LVEF at each time point (33 ± 7 %***, 29 ± 3 %*** and 31 ± 2 %***, ***p < 0. 001) as demonstrated in Fig. 1. The LVESV and LVEDV (μL) at weeks 1, 2, and 4 in the telmisartan vs. control groups (LVESV: 43 ± 31 vs. 38 ± 14; 24 ± 12 vs. 33 ± 12; and 43 ± 10 vs. 44 ± 10 and LVEDV: 63 ± 39 vs. 46 ± 15; 33 ± 16 vs. 43 ± 17, and 62 ± 14 vs. 54 ± 12) demonstrated no significant change. When comparing serially from week 1 to 4 within each group, LVESV and LVEDV in the telmisartan group demonstrated no change while the control group demonstrated a trend to increased LV volumes. The increase in LVEF was not associated with decreased afterload. Thus the increased LV output was not likely related to telmisartan’s potential to reduce blood pressure and thereby, afterload. There was no significant difference in the systemic end-diastolic pressure or tau, the isovolumic relaxation constant, as shown in Fig. 2.

### MEMRI and DEMRI scar volume

**Fig. 3:**
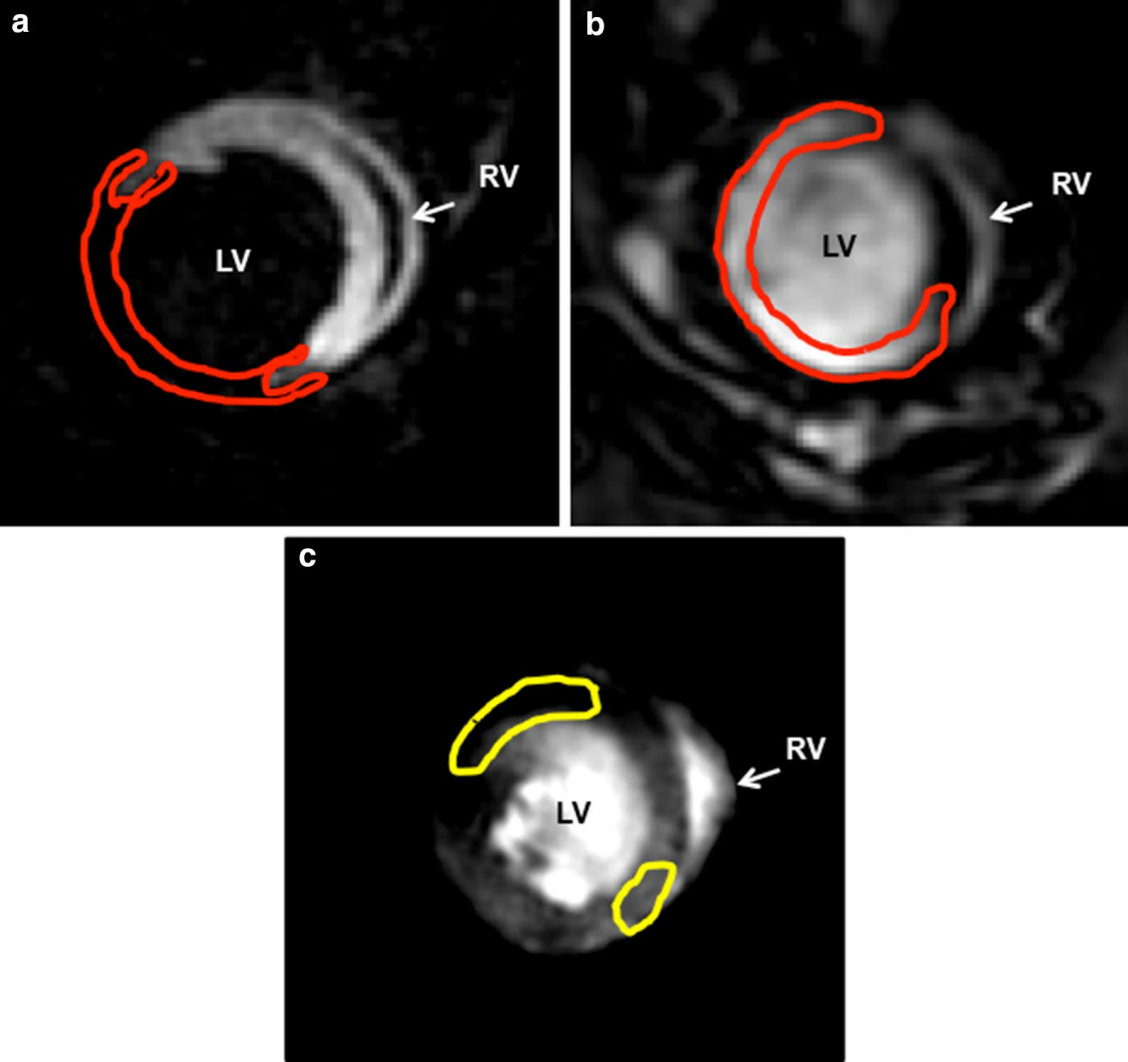
Corresponding short-axis MEMRI and DEMRI. **a** MEMRI defect representing myocardial scar (*red tracing*), **b** DEMRI enhancement depicting myocardial scar (*red tracing*), and **c** overlapping area between MEMRI and DEMRI in the border zone illustrates the viable peri-infarct region (*yellow tracing*)

**Fig. 4:**
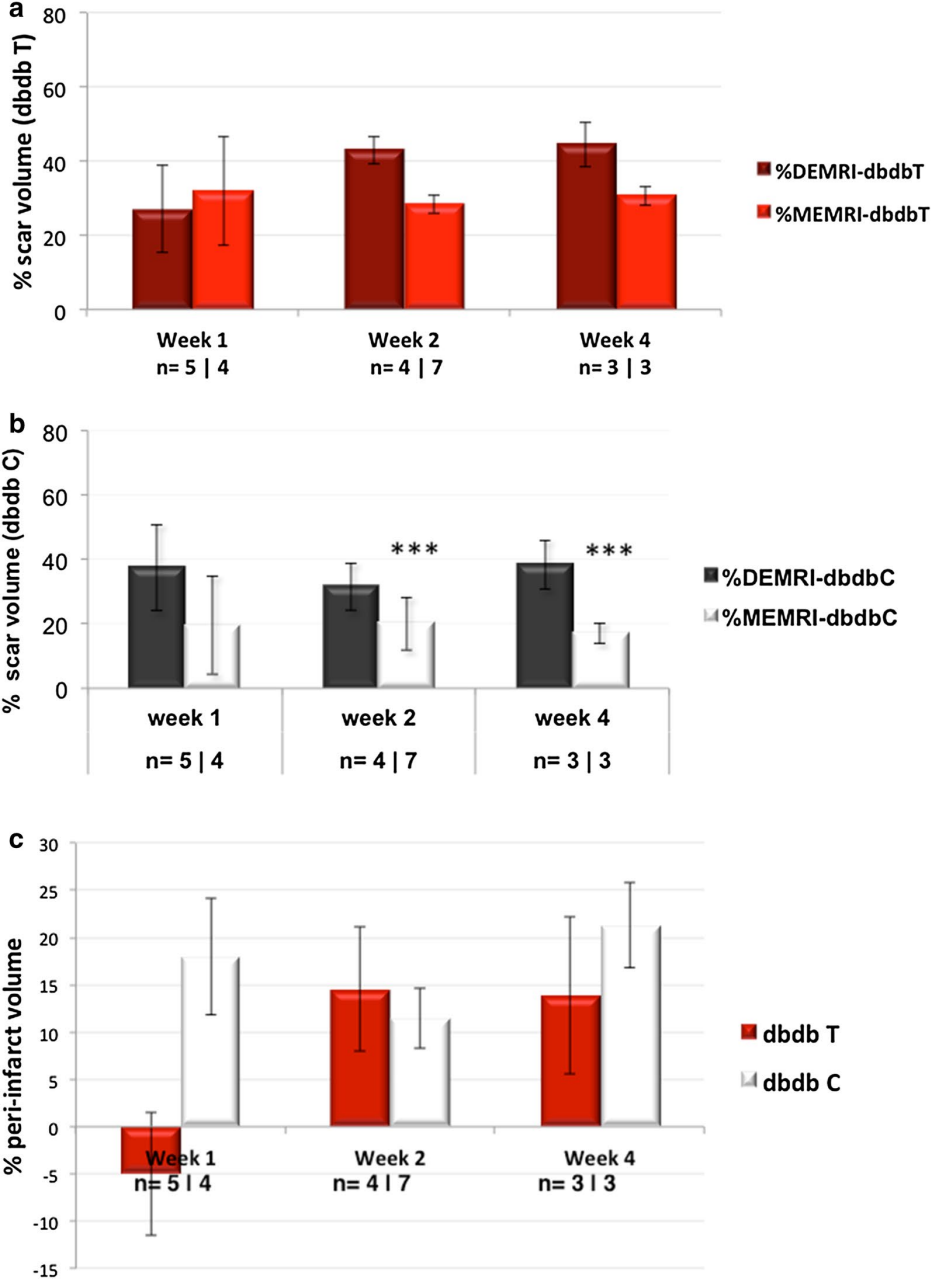
MEMRI and DEMRI enable peri-infarct volume measurements. DEMRI % scar volume does not differ significantly from the MEMRI % scar volume in the **a** telmisartan group (*red* and *dark red bars*) while **b** the DEMRI and MEMRI % scar volume difference is significant in the control (*white* and *black bars*) group for weeks 2 and 4. **c** The telmisartan group demonstrates decreased % peri-infarct volume compared to the control group at week 4

MEMRI showed reproducible and homogeneous myocardial Mn^2+^ uptake in remote (normal) areas of the myocardium. Within the anteroseptal, anterior, and anterolateral regions, MEMRI signal defect was consistently observed as demonstrated in Fig. 3a. This corresponded to the enhanced infarct zone of DEMRI images as shown in Fig. 3b. The infarct volume (% LV myocardium) at weeks 1, 2, and 4 were measured by MEMRI and DEMRI, respectively, for telmisartan and control groups (telmisartan: 32.0 ± 14.8 vs. 27.1 ± 11.7; 28.5 ± 2.5 vs. 43.0 ± 3.7; and 30.7 ± 2.6 vs. 44.5 ± 6.1; and control: 19.5 ± 15.2 vs. 37.6 ± 13.4; 20.1 ± 8.2 vs. 31.6 ± 7.3***; and 17.0 ± 3.0 vs. 38.3 ± 7.5***, ***p < 0.001). The quantitative measurement of the MEMRI defect volume revealed the scar volume was not significantly different from DEMRI measurements in the telmisartan group (Fig. 4a) while significantly lower than the corresponding DEMRI measurements at weeks 2 and 4 in the control group (Fig. 4b). This significant difference between MEMRI and DEMRI scar volumes represented the peri-infarct region (Fig. 3c). The significant difference between the smaller MEMRI defect and the larger DEMRI scar volume delineated the presence of the injured but viable (MEMRI) cardiomyocytes within the scar (DEMRI) region. This finding enabled volumetric measurement of the peri-infarct region and assessment of the potential mechanism of telmisartan in salvaging the injured myocardium. The control group at weeks 2 and 4 demonstrated the persistence of myocardial injury, resulting in the persistence of the peri-infarct region. On the other hand, telmisartan treatment attenuated this injury, minimizing the area at risk in the peri-infarct region at week 4 as illustrated in Fig. 4c.

### Myocardial fibrosis and apoptosis

**Fig. 5:**
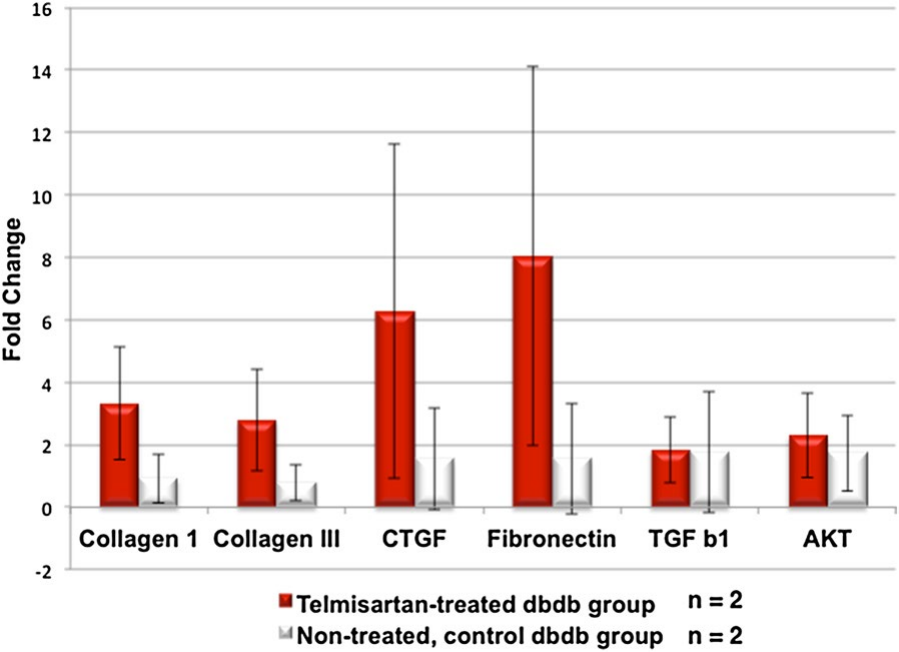
Myocardial fibrosis and apoptosis. Real-time PCR quantitative analysis of fibrotic (collagen I, collagen III, connective tissue growth factor (CTGF), TGFβ, and fibronectin) and apoptotic (thymoma viral proto-oncogene 1: Akt) genes in the injured myocardium of telmisartan and control groups. There was a trend towards increased expression of fibrotic genes, collagen I, collagen III, CTGF and fibronectin in the telmisartan group compared to the control group. *Red bar*: telmisartan group, *white bar*: control group

Expressions of molecular markers for fibrosis (collagen I, collagen III, CTGF, TGFβ, and fibronectin) and apoptosis (Akt) were measured in telmisartan and control mice heart tissue using real-time PCR. Following 4-week duration of telmisartan treatment, there was a trend towards increased expression of fibrotic genes, including collagen I, collagen III, CTGF and fibronectin (Fig. 5). Up-regulation of these genes re-establishes extracellular matrix homeostasis to attenuate left ventricular remodeling [20]. This increased trend may modulate the fibrosis of the infarcted region and salvage the injured but viable cardiomyocytes to stabilize the peri-infarct region by weeks 2 and 4.

### Hematoxylin and eosin stain and TEM of myocardial injury

**Fig. 6:**
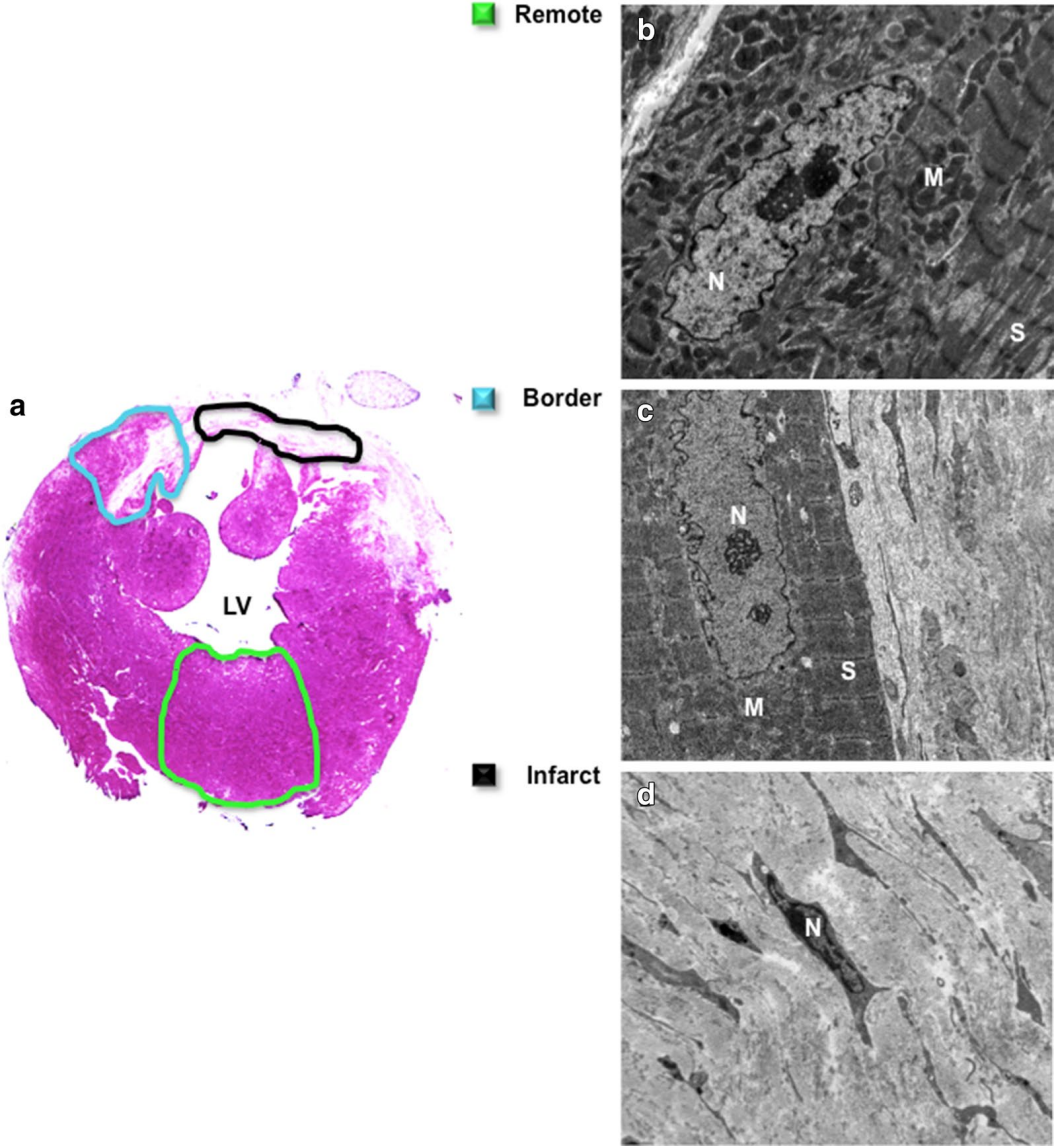
Hematoxylin and eosin stain and TEM of myocardial injury. **a** Hematoxylin and eosin stained heart with ROI of remote zone (*black line*), peri-infarct region (*blue line*), and infarct region (*green line*). **b–d** TEM images of remote, border, and infarct regions. The three regions exhibited healthy, injured but viable, and non-viable cells, respectively. *M* mitochondria, *N* nucleus, *S* sarcomeric structure

The hematoxylin and eosin stain and TEM validated the MEMRI–DEMRI assessment of the remote, peri-infarct, and infarcted myocardium (Fig. 6a–d). The hematoxylin and eosin stain demonstrated the homogeneous myocardial tissue in the remote (normal) zone. The infarcted region exhibited diffuse fibrotic and calcified appearance consistent with the infarct core. However, the peri-infarct border zone showed heterogeneous characteristics of the injured but viable cardiomyocytes. The ultrastructural characteristics of the cardiomyocytes within the remote, peri-infarct, and infarcted regions in a telmisartan-treated diabetic mouse was further characterized by TEM of nine myocardial samples from the three regions, using previously established quantitative characterization of normal nuclear, chromatin, mitochondrial, and sarcomeric structure and organization. The subcellular characteristics of the remote, peri-infarct, and infarcted zones were quantitatively analyzed (Table 1). As expected, the subcellular organelles were destroyed in the infarcted region (overall TEM score: 1.2 ± 0.2, n = 17). However, the peri-infarct region exhibited injured but viable subcellular structure, demonstrating significant difference in the score from both the remote and infarct region (3.1 ± 0.4*, p < 0.05). The remote region displayed normal ultrastructure of viable cells (4.7 ± 0.3). The TEM data indicated the presence of injured but viable cardiomyocytes with intact subcellular organelles, which validated the MEMRI–DEMRI signal consistent with the presence of the injured but viable cardiomyocytes in the peri-infarct region.

**Table 1:** Quantitative summary of the different cellular structures in the remote, border and infarct zone in a 4-week telmisartan-treated diabetic mouse (* p < 0.05)

Cardiomyocyte ultrastructure score by tissue electron microscopy				
Cell structure	Characteristic feature	Remote	Border	Infract
		Mean ± SD	Mean ± SD	Mean ± SD
Nuclei	Notched/furrowed membrane	4.5 ± 0.7	3.9 ± 1.0	1.1 ± 0.4
	Homogenous chromatin granules	4.8 ± 0.4	4.3 ± 0.8	1.3 ± 0.5
	Chromatin accumulated along nuclear membrane	5.0 ± 0.0	4.2 ± 0.8	1.9 ± 0.6
	Chromatin clots within nucleus	4.8 ± 0.4	4.3 ± 0.8	1.3 ± 0.5
	Dense chromatin	4.9 ± 0.3	4.3 ± 0.9	1.3 ± 0.5
	Dark chromatin finely structured	4.9 ± 0.3	4.5 ± 0.7	1.2 ± 0.4
Mitochondria	Dense peri-nuclear accumulation	4.6 ± 0.5	3.8 ± 1.4	1.2 ± 0.4
	Fine filaments/glycogen granules between nucleus & mitochondria	4.8 ± 0.4	4.1 ± 1.0	1.3 ± 0.5
	Destroyed cristea	4.8 ± 0.4	3.7 ± 1.0	1.1 ± 0.4
	Few mitochondria near nucleus	4.8 ± 0.4	3.9 ± 1.5	1.1 ± 0.3
	Mitochondria isolated in niche	4.7 ± 0.6	4.1 ± 1.4	1.1 ± 0.4
Myofibrils	Myofibrils aligned in one row	4.8 ± 0.5	3.9 ± 0.8	1.0 ± 0.2
	T-tubules contain basal lamina	4.9 ± 0.4	4.7 ± 0.6	1.1 ± 0.3
	Myofibrils are contracted	4.8 ± 0.4	4.2 ± 0.9	1.0 ± 0.3
	Z-line disruption	4.9 ± 0.3	4.1 ± 0.7	1.0 ± 0.2
	Lipid droplets between ruptured myofibrils	4.3 ± 0.5	3.6 ± 1.1	1.2 ± 0.4
	Collagen	4.0 ± 0.9	3.1 ± 0.9	1.0 ± 0.2
	*Overall TEM scores*	*4.7* ± *0.3*	*3.1* × 0.4*	*1.2* × *0.2*

*p < 0.05

### Flow cytometry for circulating CD34 + cells

**Fig. 7:**
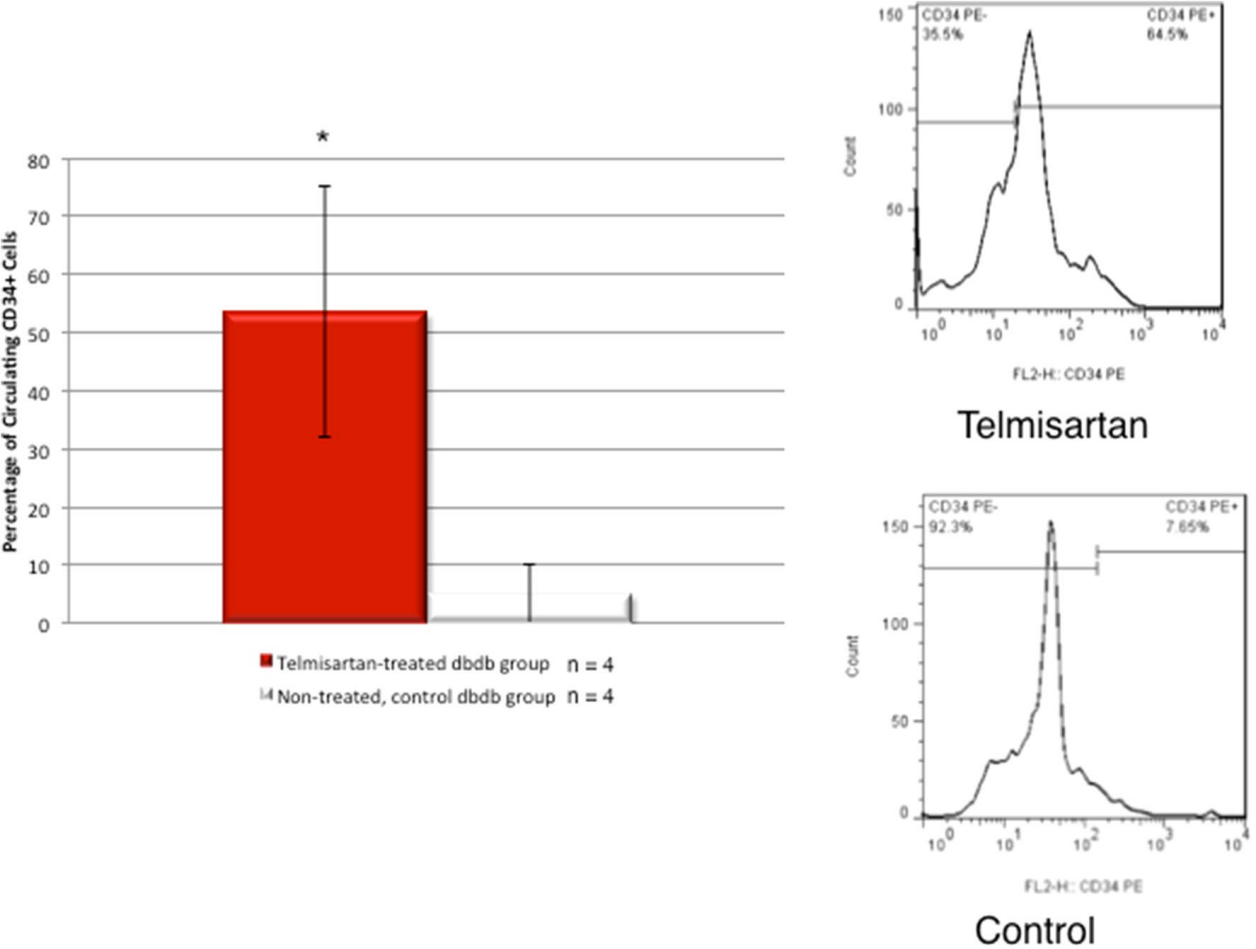
Flow cytometry of circulating CD34 + cells. The telmisartan group demonstrates significantly increased population of circulating CD34 + cells compared to the control group (*p < 0.05)

Flow cytometry performed on whole blood demonstrated a significant increase in circulating CD34 + cells in the telmisartan-treated vs. control mice (53.5 %* vs. 5.0 %, *p < 0.05), which constitute the EPCs, as demonstrated in Fig. 7 [21–23].

## Discussion

The current study confirmed the benefit of telmisartan in improving the cardiac function in the diabetic murine myocardial injury model. Dual-contrast MRI tracked the therapeutic effects of telmisartan on the injured but viable myocardium in the peri-infarct region. Telmisartan treatment demonstrated significant attenuation of the peri-infarct region with resultant improved LV function. Afterload reduction did not contribute to improved LV function, signifying the importance of the role of the injured but viable cardiomyocytes in the peri-infarct region to restore cardiac function. These data, validated by TEM, supported the study’s finding that the underlying mechanism of telmisartan in restoring the myocardium is predominantly due to the significant salvage of the peri-infarct region. In addition, high morbidity and mortality of advanced heart failure are associated with ventricular arrhythmia and LV remodeling in the peri-infarct region [24]. The peri-infarct region has been recognized as an important substrate to trigger ventricular arrhythmias and revascularization of the ischemic peri-infarct region have been shown to lower the incidence of ventricular arrhythmias and LV remodeling [25].

The renin-angiotensin system plays a critical role in the pathophysiology of cardiovascular disease [26]. Telmisartan shows high affinity for the AT_1_ receptor with the longest half-life (24 h) of any ARB [27]. Activation of AT_1_ by angiotensin II leads to molecular and cellular events involved in endothelial damage and progression of atherosclerosis, which is attenuated by telmisartan. Endothelial regeneration is not only accomplished by resident endothelial cells but also by EPCs [28]. Poor glycemic control in the diabetic population correlates with significantly decreased circulating EPCs [29]. Impaired function and quantity of circulating EPCs are associated with poor cardiovascular outcomes in patients with coronary artery disease [28, 30]. Endtmann et al. [28] demonstrated that angiotensin II consistently decreased the proliferation of EPCs in vitro and in vivo through the activation of AT_1_. As suggested by the flow cytometry data, telmisartan increased the number of circulating EPCs and may have led to an improved physiologic function of the endothelium and neovascularization in the peri-infarct region. Although afterload reduction effects of ARBs can modulate the vascular phenotype to preserve normal flow-mediated vasodilation, the study did not demonstrate any significant afterload reduction with telmisartan.

In addition to blocking the renin-angiotensin system, telmisartan acts as a selective modulator of PPARγ, a central regulator of insulin and glucose metabolism and a well-known target of insulin-sensitizing drugs used to treat type 2 diabetes mellitus [9]. Toyama et al. [30] obtained the first evidence that partial PPARγ activity of telmisartan contributed to greater vascular protective effects of telmisartan than losartan in obese and diabetic mice. The underlying mechanism involved the normalization of Akt/eNOS cascade and anti-inflammatory effect due to the suppression of NFκB and TNFα. The dual effects of telmisartan in selective antagonism of the AT_1_ receptor and modulation of PPARγ may have ameliorated the diabetic vascular complications and salvaged the injured myocardium.

Finally, up-regulation of the genes specific to the secretion of collagens and CTGF can act to re-establish extracellular matrix homeostasis and ultimately, inhibit cardiac fibrosis to attenuate left ventricular chamber dilation post myocardial infarction [20]. CTGF, in particular, is a multifunctional growth and differentiation factor and it can promote chemotaxis, migration, adhesion, proliferation, differentiation and/or extracellular matrix formation [31]. Its specific effects depend on the type of cell with which it interacts. CTGF has been shown to bind to IGF and VEGF to modulate their actions and promote angiogenesis [32]. This increased trend of collagen I, collagen III, CTGF and fibronectin may explain the underlying mechanism of telmisartan in modulating the fibrosis of the infarcted region while salvaging the injured but viable cardiomyocytes to stabilize the peri-infarct region by weeks 2 and 4.

In this study, the dual contrast MEMRI–DEMRI strategy defined the peri-infarct region to be the border zone of the scar where there is significant viable myocardium by MEMRI. This novel MRI technique was employed to elucidate the potential underlying mechanism of telmisartan in restoring the myocardial dysfunction after myocardial injury. The ultrastructural analysis of the telmisartan group by TEM demonstrated the significantly increased presence of viable but injured cardiomyocytes. The dual MEMRI–DEMRI contrast technique demonstrated significant attenuation of the peri-infarct region by telmisartan when compared to the control group. This significant reduction may underlie the improved myocardial function of telmisartan treatment.

In conclusion, telmisartan demonstrated significant functional restoration of the injured diabetic murine myocardium by reducing the peri-infarct region, which was possibly mediated by increased neovascularization. The dual-contrast MEMRI–DEMRI technique enabled identification and systematic evaluation of at-risk but viable peri-infarct myocardium.

## Limitations

The major limitation of the present study is the higher mortality rate of our diabetic mouse model with myocardial injury. With repeated anesthesia necessary for serial studies and increased morbidity with this diabetic mouse model that was not receiving any diabetic therapies, this resulted in fewer mice that survived at the end of the study. Though we still identified significant trends, we believe data from more surviving mice would have resulted in identifying further meaningful trends. Another limitation was that DEMRI and MEMRI were performed on consecutive days due to adequate clearance of contrast necessary in between scans. In a prior study, no significant difference in infarct size has been shown to occur with repeated imaging within 24–48 h [33]. However, ideally, both DEMRI and MEMRI would be performed in the same scan to limit the potential for differences to occur due to timing.

## Abbreviations

ACE: angiotensin-converting enzyme
ARB: angiotensin receptor blocker
DEMRI: delayed enhancement MRI
EPC: endothelial progenitor cell
LAD: left anterior descending coronary artery
LV: left ventricle
LVEDV: LV end-diastolic volume
LVEF: LV ejection fraction
LVESV: LV end-systolic volume
MEMRI: manganese-enhanced MRI
PPARγ: peroxisome proliferator-activated receptor gamma
TEM: transmission electron microscopy
